# PHYSICAL ACTIVITY BARRIERS AND INTENTIONS OF ALZHEIMER’S DISEASE (AD) AND RELATED DEMENTIAS (AD/ADRD) CAREGIVERS

**DOI:** 10.1093/geroni/igad104.3525

**Published:** 2023-12-21

**Authors:** Andrew Pickett, Rebecca Krukowski, Nicole Werner

**Affiliations:** Indiana University-Bloomington, Bloomington, Indiana, United States; University of Virginia, School of Medicine, Charlottesville, Virginia, United States; Indiana University School of Public Health-Bloomington, Bloomington, Indiana, United States

## Abstract

Caregivers of people living with AD/ADRD often experience negative biopsychosocial health outcomes and have reduced capacity to engage in healthy behaviors (e.g., exercise). Physical activity is an established intervention for promoting physical and mental health across many populations. This research explored physical activity intentions and barriers among AD/ADRD caregivers, as well as potential relationships between physical activity intentions, cohabitation status, and caregiver/care recipient relationship. Data were collected via online survey from family caregivers of people living with AD/ADRD (n=117). The sample was predominantly women (n=89; 76.1%), white (n= 89, 76.1%), and averaged 55.3 (SD= 16.45) years old. Participants averaged 56.31 (SD= 49.15) hours per week on care responsibilities. Caregivers expressed strong desire to be more active (M=4.42 / 5; SD= .71) and that caregiving demands were a strong barrier (M=3.85 / 5; SD= 1.15). Significant differences were observed between caregivers who lived, or did not live with, care recipients across both physical activity intention (p= .022) and burden perceptions (p=.002), measured by the short form Burden Scale for Family Caregivers (BSFC-SF). No differences were observed based on caregiver-care recipient relationship (e.g., parent, partner). Primary themes among open-ended responses to physical activity barriers included: lack of time and energy, inability to leave care recipient, and competing responsibilities. Our findings suggest a need for behavioral interventions to facilitate physical activity among AD/ADRD caregivers, particularly those who live with their care recipient. Social network interventions that leverage social connections to support caregivers may increase physical activity by reducing barriers associated with care responsibilities.

